# Screening, prevention and early diagnosis of preeclampsia: need for an updated protocol in Peru

**DOI:** 10.17843/rpmesp.2024.413.13793

**Published:** 2024-07-30

**Authors:** Rommy H. Novoa, Carlos Pérez-Aliaga, Jose E. Castañeda-Apolinario, Alexandra I. Ramírez-Moreno, Luis Meza-Santibañez

**Affiliations:** 1 National Maternal Perinatal Institute. Lima, Peru. National Maternal Perinatal Institute Lima Peru; 2 Alberto Hurtado Faculty of Medicine, Universidad Peruana Cayetano Heredia. Lima, Peru. Universidad Peruana Cayetano Heredia Alberto Hurtado Faculty of Medicine Universidad Peruana Cayetano Heredia Lima Peru; 3 San Fernando Faculty of Medicine, Universidad Nacional Mayor de San Marcos. Lima, Peru. Universidad Nacional Mayor de San Marcos San Fernando Faculty of Medicine Universidad Nacional Mayor de San Marcos Lima Peru

To the Editor. Preeclampsia is a multisystem disorder of gestation characterized by placental perfusion impairment resulting in maternal vascular endothelial injury causing blood pressure elevation ≥140/90 mm Hg and multiorgan injury ^1^^,^^2^. In addition, it can cause fetal growth restriction and intrauterine death. It constitutes the second cause of maternal death after hemorrhage in Peru and the world ^3^^,^^4^, and is a significant cause of long-term morbidity in the mother and fetus ^2^.

Delivery is the treatment for preeclampsia. However, the optimal timing should take into account the balance between decreasing disease progression and thus maternal risks, and decreasing neonatal complications due to prematurity. Current management, aimed at achieving the best maternal and perinatal outcomes, is focused on prediction, prevention and early diagnosis ^1^. Validated screening strategies are based on the evaluation of a combination of clinical risk factors, serum markers such as placental growth factor (PIGF) and pregnancy-associated placental protein A (PAPA-A) and Doppler flow analysis of maternal uterine arteries ^1^^,^^2^, before 14 weeks of pregnancy. A high-risk screening (>1/150) determines treatment with aspirin, 100 to 150 mg, before 16 weeks of gestation. This is the only preventive medication backed by solid scientific evidence, that reduces the risk of developing preeclampsia before 37 weeks of pregnancy up to 62% ^1^. Likewise, early diagnosis can be achieved by identifying angiogenic imbalance, since circulating levels of soluble fms-like tyrosine kinase 1 (sFlt1), an anti-angiogenic factor, are markedly increased in women with preeclampsia, and PIGF is decreased. This imbalance precedes the onset of clinical signs of preeclampsia and correlates with disease severity ^5^.

**Figure 1:**
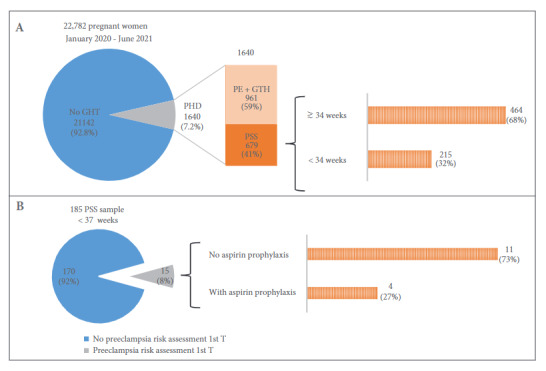
Analysis of births at the National Maternal Perinatal Institute in Lima, Peru 2020-2021. (A) Prevalence of pregnancy hypertensive disorders according to severity. (B) Risk assessment in the first trimester of pregnancy and application of aspirin prophylaxis in a sample of 185 women with preeclampsia with signs of severity (PSS). PHD: pregnancy hypertensive disorder, PE: preeclampsia, GHT: gestational hypertension.

We assessed the prevalence of pregnancy hypertensive disorders (PHD) in all patients (N=22782) from the Instituto Nacional Materno Perinatal (INMP), a high-complexity referral center in gynecological-obstetric pathology in Peru, between January 2020 and June 2021 (Figure 1A). The overall prevalence of PHD was 7.2% (1640/22782) and of these cases, 41.4% (679/1640) presented preeclampsia with signs of severity, being 32.0% (215/679) of them far from term (less than 34 weeks of gestation). In addition, we evaluated a sample of 185 women with severe preterm preeclampsia (less than 37 weeks) of whom 87.4% were referred for care of pregnancy complications during the second and third trimester. Only 8% (15/185) of the patients had a first-trimester preeclampsia risk assessment, and of these, 27% (4/15) received aspirin prophylaxis (Figure 1, B). This study complied with the ethical principles in research and had the ethical approval of the Institutional Ethics Committee (006-2022-CIEI/INMP).

PSS: Preeclampsia with signs of severity PHD: Pregnancy hypertensive disorder PE: preeclampsia GHT: gestational hypertension.

The Ministry of Health through its 2013 Technical Health Standard for Comprehensive Maternal Health Care (NTS No. 105-MINSA/DGSP-V.01), protocols prenatal control at all levels of care in the country. This standard establishes refocused prenatal care, which consists of surveillance and comprehensive evaluation of pregnant women before 14 weeks to provide timely care that allows detection of risk factors and management of pregnancy complications. The aim is to achieve a minimum of 6 prenatal care visits as a synonym of quality prenatal care ^6^. However, there is no modern protocol for risk assessment of preeclampsia, nor any national policy for prevention or early diagnosis in accordance with the current scientific evidence that guides modern obstetrics. Moreover, reports show that the implementation of a first trimester screening program for preeclampsia and early intervention with aspirin in women at high risk is associated with cost savings in the health care system. Thus, by preventing a significant number of cases of preeclampsia with inexpensive measures such as aspirin and ultrasound, compared to managing the disease per se, the care of women with severe preeclampsia and premature neonates in Intensive Care Units, which generate extremely high costs to the health system and can be associated with permanent disabilities, would be avoided ^7^. In line with this, the INMP published in 2023 a complete protocol on prediction, prevention, diagnosis and treatment of hypertensive disorders within the update of its guidelines and procedures in obstetrics ^8^, which should be a model for updating the national protocol.

In conclusion, we report the lack of preeclampsia risk assessment and the use of aspirin as prophylaxis in patients who developed severe preterm preeclampsia. In this context, the absence of a protocol for prediction, prevention and early diagnosis in the Technical Standard for maternal care in force in Peru since 2013 stands out. An update of the technical standard and establishing a national protocol aimed at universal screening of pregnant women is urgently required to focus preventive measures on those at high risk and reduce maternal and perinatal morbidity and mortality due to preeclampsia in Peru.
