# Supplementary material for: Screening, prevention and early diagnosis of preeclampsia: need for an updated protocol in Peru
Source: Rev Peru Med Exp Salud Publica. 2024 Jul 30;41(3):321–3. doi: 10.17843/rpmesp.2024.413.13793 (PMC11495920; doi:10.17843/rpmesp.2024.413.13793)
Supplement: Translation

# Translation: Tamizaje, prevención y diagnóstico temprano de preeclampsia: necesidad de un protocolo actualizado en el Perú

**Authors:** Rommy H. Novoa, Carlos Pérez-Aliaga, Jose E. Castañeda-Apolinario, Alexandra I. Ramírez-Moreno, Luis Meza-Santibañez

**Affiliations:** 1 Instituto Nacional Materno Perinatal, Lima, Perú.; 2 Facultad de Medicina Alberto Hurtado, Universidad Peruana Cayetano Heredia, Lima, Perú.; 3 Facultad de Medicina de San Fernando, Universidad Nacional Mayor de San Marcos, Lima, Perú.

Sr Editor. La preeclampsia es un trastorno multisistémico de la gestación que se caracteriza por diversos niveles de afectación de la perfusión placentaria que producen una injuria endotelial vascular materna ocasionando elevación de la presión arterial ≥140/90 mm Hg e injuria multiorgánica ^1^^,^^2^. Además, puede ser causa de restricción del crecimiento fetal y muerte intraútero. Constituye la segunda causa de muerte materna después de la hemorragia en el Perú y el mundo ^3^^,^^4^, y es una causa significativa de morbilidad a largo plazo en la madre y el feto ^2^.

El tratamiento de la preeclampsia es el parto. Sin embargo, el momento óptimo debe tener en cuenta el balance entre disminuir la progresión de la enfermedad y con ello, los riesgos maternos, y lograr las menores complicaciones neonatales por prematuridad. El manejo actual, para lograr los mejores desenlaces maternos y perinatales, está enfocado en la predicción, prevención y diagnóstico temprano ^1^. Las estrategias validadas de tamizaje están basadas en la evaluación de la combinación de factores de riesgo clínicos, marcadores séricos como el factor de crecimiento placentario (PIGF) y la proteína A placentaria asociada al embarazo (PAPA-A) y el análisis del flujo Doppler de las arterias uterinas maternas ^1^^,^^2^, antes de las 14 semanas de embarazo. Un tamizaje que resulte de alto riesgo (>1/150) determina la administración de aspirina, entre 100 a 150 mg, antes de las 16 semanas de gestación. Este es el único medicamento preventivo demostrado con evidencia científica sólida, logrando una reducción de hasta 62% del riesgo de desarrollar preeclampsia antes de las 37 semanas de embarazo ^1^. Asimismo, el diagnóstico temprano se puede lograr con la identificación del desbalance angiogénico, ya que los niveles circulantes de tirosina quinasa 1 soluble similar a fms (sFlt1), un factor anti angiogénico, está notablemente aumentados en mujeres con preeclampsia, y el PIGF está disminuido. Este desbalance precede a la aparición de signos clínicos de preeclampsia y se correlaciona con la gravedad de la enfermedad ^5^.

Realizamos una evaluación de la prevalencia de los trastornos hipertensivos del embarazo (THE) en todas las pacientes atendidas (N=22782) en el Instituto Nacional Materno Perinatal (INMP), centro de mayor complejidad y de referencia nacional en patología gineco-obstétrica en el Perú, entre enero 2020 y junio 2021 (Figura 1A). La prevalencia general de THE fue de 7,2% (1640/22782) y de ellas, el 41,4% (679/1640) presentaron preeclampsia con signos de severidad siendo el 32,0% (215/679) de ellas lejos del término (con menos de 34 semanas de gestación). Además, evaluamos una muestra de 185 mujeres con preeclampsia severa pretérmino (menor a 37 semanas) de las cuales, el 87,4% fueron referidas para atención de complicaciones del embarazo en el segundo y tercer trimestre. Solo el 8% (15/185) de las pacientes tuvo una evaluación del riesgo de preeclampsia en el primer trimestre, y de ellas, el 27% (4/15) recibieron profilaxis con aspirina (Figura 1, B). Este estudio siguió los principios éticos en investigación y tuvo la aprobación ética del Comité de Ética institucional (006-2022-CIEI/INMP).

**Figura 1:** Análisis de nacimientos atendidos en el Instituto Nacional Materno Perinatal en Lima, Perú 2020-2021. (A) Prevalencia de trastornos hipertensivos del embarazo según severidad. (B) Evaluación de riesgo en el primer trimestre del embarazo y aplicación de profilaxis con aspirina en una muestra de 185 mujeres con preeclampsia con signos de severidad (PES). THE: trastorno hipertensivo del embarazo, PE: preeclampsia, HTG: hipertensión gestacional.

PES: Preeclampsia con signos de severidad THE: Trastorno hipertensivo del embarazo PE: preeclampsia HTG: hipertensión gestacional

El Ministerio de Salud mediante su Norma Técnica de Salud para la Atención Integral de Salud Materna del año 2013 (NTS Nº 105-MINSA/DGSP-V.01), protocoliza el control prenatal en todos los niveles de atención en el país. Esta norma establece la atención prenatal reenfocada que consiste en vigilancia y evaluación integral de las gestantes antes de las 14 semanas para brindar atención oportuna que permita detección de factores de riesgo y manejo de complicaciones del embarazo. El objetivo es lograr un mínimo de 6 atenciones prenatales como sinónimo de calidad del control prenatal ^6^. Sin embargo, no presenta ningún protocolo moderno de evaluación de riesgo de preeclampsia, así como ninguna política nacional de prevención ni diagnóstico temprano acorde con la evidencia científica actual que guía la obstetricia moderna. Más aún, está reportado que la implementación de un programa de tamizaje del primer trimestre para preeclampsia y la intervención temprana con aspirina en mujeres con alto riesgo, está asociado al ahorro de costos en el sistema de salud. Así, al prevenir un significativo número de casos de preeclampsia con medidas no tan costosas como administración de aspirina y realización de ecografía, comparado con manejar la patología instalada, se evitaría la atención de mujeres con preeclampsia severa y neonatos prematuros en Unidades de Cuidados Intensivos que generan costos sumamente altos al sistema de salud y que pueden asociarse a discapacidades permanentes ^7^. En línea con ello, el INMP en el año 2023, publicó un protocolo completo sobre predicción, prevención, diagnóstico y tratamiento de trastornos hipertensivos dentro de la actualización de sus guías y procedimientos en obstetricia ^8^, que debería ser modelo de la actualización del protocolo nacional.

En conclusión, se reporta una baja aplicación de la valoración de riesgo de preeclampsia y el uso de aspirina como profilaxis en pacientes que desarrollaron preeclampsia severa pretérmino. En este contexto, se destaca la ausencia de un protocolo de predicción, prevención y diagnóstico temprano en la Norma Técnica de atención materna vigente en el Perú desde el 2013. Se requiere de manera urgente la actualización de la norma técnica y el establecimiento de un protocolo nacional que logre el tamizaje universal de las gestantes para focalizar las medidas preventivas en aquellas de alto riesgo y disminuir la morbimortalidad materna y perinatal por preeclampsia en el país.
